# RISE for recovery after acute whiplash injury: protocol for a randomised feasibility trial of a clinician-supported SMS-based behavioural intervention

**DOI:** 10.1136/bmjopen-2026-123039

**Published:** 2026-09-11

**Authors:** Rachel A Elphinston, Jessica Formosa, Nigel Armfield, Anton H van der Vegt, Claire E Ashton-James, Katherine Brain, Lisa Buckley, Christopher Papic, Asaduzzaman Khan, Chloe-Emily Eather, Jenna Liimatainen, Andrew Hobbins King, Kim Hansen, Andrew Staib, Gaylene M Smith, Tina Chen, Karen Wright, Elizabeth Garrad, Michele Sterling

**Affiliations:** 1 RECOVER Injury Research Centre, The University of Queensland, Brisbane, Queensland, Australia; 2 Centre for Innovation in Pain and Health Research (CIPHeR), The University of Queensland, Brisbane, Queensland, Australia; 3 STARS Education and Research Alliance, Surgical Treatment and Rehabilitation Service (STARS), The University of Queensland and Metro North Health, Brisbane, Queensland, Australia; 4 NHMRC Centre of Research Excellence: Better Health Outcomes for Compensable Injury, The University of Queensland, Brisbane, Queensland, Australia; 5 Centre for Health Services Research, The University of Queensland, Brisbane, Queensland, Australia; 6 Sydney Medical School, Faculty of Medicine and Health, The University of Sydney, Sydney, New South Wales, Australia; 7 College of Health Medicine and Wellbeing, The University of Newcastle, Newcastle, New South Wales, Australia; 8 MAIC/UniSC Road Safety Research Collaboration, University of the Sunshine Coast, Sippy Downs, Queensland, Australia; 9 School of Health and Rehabilitation Sciences, Faculty of Health Medicine and Behavioural Sciences, The University of Queensland, Brisbane, Queensland, Australia; 10 Trauma Service and Department of Emergency Medicine, Sunshine Coast Hospital and Health Service, Nambour, Queensland, Australia; 11 Virtual Emergency Department, Metro North Hospital and Health Service, Herston, Queensland, Australia; 12 Emergency Department, Princess Alexandra Hospital, Metro South Hospital and Health Service, Woolloongabba, Queensland, Australia

**Keywords:** PAIN MANAGEMENT, Musculoskeletal disorders, Psychological Stress, Randomized Controlled Trial

## Abstract

**Introduction:**

Whiplash-associated disorders (WADs) are the most common and costly consequence of road traffic injury (RTI). Providing early and effective treatment is a healthcare priority; however, current guideline-based care which focuses on education and physiotherapy-led exercise does not routinely address the psychosocial effects of trauma, and access to early integrated treatment is limited. This study aims to evaluate the feasibility of delivering and evaluating recovery, information, support and empowerment (RISE), a co-designed short message service-based intervention integrating psychosocial and exercise/activity support for individuals with acute whiplash injury who are at risk of poor recovery in a randomised feasibility trial. Secondary aims are to explore preliminary changes in candidate clinical outcomes and opportunities for intervention personalisation and future implementation.

**Methods and analysis:**

This mixed-methods randomised controlled feasibility trial with a nested process evaluation will recruit 50 participants (>18 years) with WAD grade II/III who are identified at medium to high risk of poor recovery and within 12 weeks of RTI from Queensland emergency departments and the community. Participants will be randomised in a 1:1 ratio to receive either the RISE intervention (tailored, daily messaging+optional brief clinician support session/s) or an active control (general health-related text messages plus access to an online resource, My Whiplash Navigator). Feasibility outcomes, including recruitment and retention rates, data collection (including wearable-derived and economic data) and intervention uptake will be descriptively summarised and evaluated against pre-defined traffic light progression criteria. Acceptability will be examined using validated questionnaires and purpose-built items, qualitative interviews with intervention participants and focus groups with patients, clinicians and funders. Secondary aims will be addressed through preliminary estimates of between-group differences in patient-reported outcomes with 95% CIs and exploratory analysis of multimodal data (eg, wearable-derived physiological data, intervention engagement metrics) using a large language model where possible to inform future personalisation and implementation planning.

**Ethics and dissemination:**

This trial is approved by the Townsville Hospital Human Research Ethics Committee (HREC/QTHS/121395) and ratified by the Human Research Ethics Committee at The University of Queensland, which is the study sponsor (2025/HE002491). Results will be published in peer-reviewed journals and presented at conferences and professional meetings.

**Trial registration:**

ACTRN12626000506392; date registered: 23 April 2026; https://www.anzctr.org.au/Trial/Registration/TrialReview.aspx?id=390706&isReview=true

**STRENGTHS AND LIMITATIONS OF THIS STUDY**

- First of its kind feasibility evaluation of a short message service-based intervention to support recovery after whiplash injury following road traffic crashes.
- Participant-masked and assessor-blinded randomised controlled trial design to assess feasibility outcomes against a pre-specified traffic-light framework, supporting transparent decision-making regarding progression to a larger definitive trial.
- A nested qualitative study enables triangulation of quantitative feasibility data with in-depth participant perspectives on intervention experience and delivery.
- The recovery, information, support and empowerment intervention was co-designed with patients, clinicians and insurance representatives, enhancing methodological relevance, acceptability and implementation fidelity.
- The intervention targets individuals at medium to high risk of poor recovery within 12 weeks of injury, which may limit methodological generalisability to later stages of recovery.

## Introduction

 Providing early and effective treatment for whiplash-associated disorders (WAD), the most common and costly consequence of road traffic injury (RTI), is a healthcare priority. Currently, clinical guidelines for WAD treatment recommend education and exercise/activity in the first 12 weeks after injury.^1^ However, these approaches demonstrate only modest and short-term benefits.^2–4^ More than one-third of people, particularly those at high risk of poor recovery, do not fully recover and continue to experience persistent pain and disability beyond the acute phase.^5^ Ongoing symptoms are frequently accompanied by psychological comorbidities, including post-traumatic stress disorder, which further complicate recovery trajectories.^6^

Targeted approaches that address the psychological effects of trauma and modifiable psychological risk factors alongside guideline-based care are increasingly recognised as important for improving recovery and well-being following RTI.^7^ There is substantial evidence for the role of psychological factors in recovery from WAD^8–10^ as well as the broader psychological impacts associated with the lived experience of RTI.^11 12^ Notably, an early integrated psychological and physiotherapy exercise intervention targeting individuals at risk of poor recovery has been shown to improve pain-related disability compared with exercise alone in patients with acute WAD, with effects maintained at 12 months.^13^ Secondary analysis of this clinical trial indicated that reductions in stress symptoms and improvements in pain-related coping represented key pathways through which this intervention exerted its effects on disability.^14^ However, timely access to early targeted care remains limited, and there is a need for complementary approaches that are low-intensity, cost-effective and scalable within routine clinical pathways, including compensation-based healthcare systems.

Digital health interventions offer an opportunity to deliver early, scalable and accessible support following injury. One promising approach is the use of short message service (SMS)-based interventions, which are increasingly used in healthcare settings and require minimal digital literacy.^15^ SMS interventions have been shown to be effective and acceptable in promoting behaviour change, improving self-management and supporting recovery across a range of chronic health conditions (eg,^16 17^). Emerging evidence suggests that SMS interventions may also be beneficial in the management of musculoskeletal pain; however, there are few high-quality studies in this area,^18^ and limited research has incorporated co-design methods to tailor SMS interventions to patient and clinician needs, preferences and lived experience.^19^ To date, the potential of SMS-based interventions to support recovery in individuals with WAD and the potential to integrate digital technologies, such as wearable devices and human-centred artificial intelligence to enhance personalisation and optimisation of early integrated care has yet to be examined.

The primary aim of this study is to evaluate the feasibility of delivering and evaluating recovery, information, support and empowerment (RISE), a co-designed SMS-based early intervention to improve recovery for individuals with WAD who are at risk of poor recovery within the first 12 weeks following a RTI, evaluated in a randomised controlled feasibility trial. The RISE intervention includes daily personalised text messages and optional brief psychologist and physiotherapy support. It will be compared with an active control condition comprising general health-related text messages plus access to an online resource, *My Whiplash Navigator,* to facilitate participant blinding. Feasibility will be assessed in terms of recruitment, retention, intervention engagement, collection of wearable-derived and cost data and acceptability. Pre-defined criteria, interpreted alongside the qualitative process measures from patients, clinicians and funders, will guide decisions regarding progression to a future definitive trial. Secondary aims are to: (1) explore possible preliminary changes in candidate patient-reported clinical outcomes comparing RISE with an active control condition and (2) explore the feasibility of analysing multimodal data, including wearable-derived physiological metrics (eg, activity and sleep), patient-reported outcomes and intervention engagement data using a large language model to generate insights to inform future intervention personalisation and optimisation. Findings from this study will inform the need for and design of a future definitive trial.

## Methods and analysis

### Study design and setting

This is a mixed-methods randomised controlled feasibility trial with blinded outcome assessment. Fifty adults with acute WAD (<12 weeks post-injury) who are at medium or high risk of poor recovery will be randomly allocated 1:1 to either the RISE intervention or active control (25 per group^20^). Participants will be recruited through three Queensland-based metropolitan hospital emergency departments (ED), including Sunshine Coast University Hospital, Princess Alexandra Hospital (PAH) and Metro North Virtual Emergency Care Services (VECS). Recruitment will also occur through community channels, including social media, digital marketing and local clinics, to ensure the target sample size is reached.

Participant blinding will be supported by providing both groups with SMS contact without disclosing group allocation; however, full participant blinding may not be possible given differences in intervention intensity and content. Outcomes will be measured at baseline and at 6-week and 3-month post-randomisation. Qualitative methods including focus groups and individual semi-structured interviews will examine patient acceptability of the intervention and explore perspectives of patients, clinicians and funders on its potential future optimisation and implementation.

**Figure 1:**
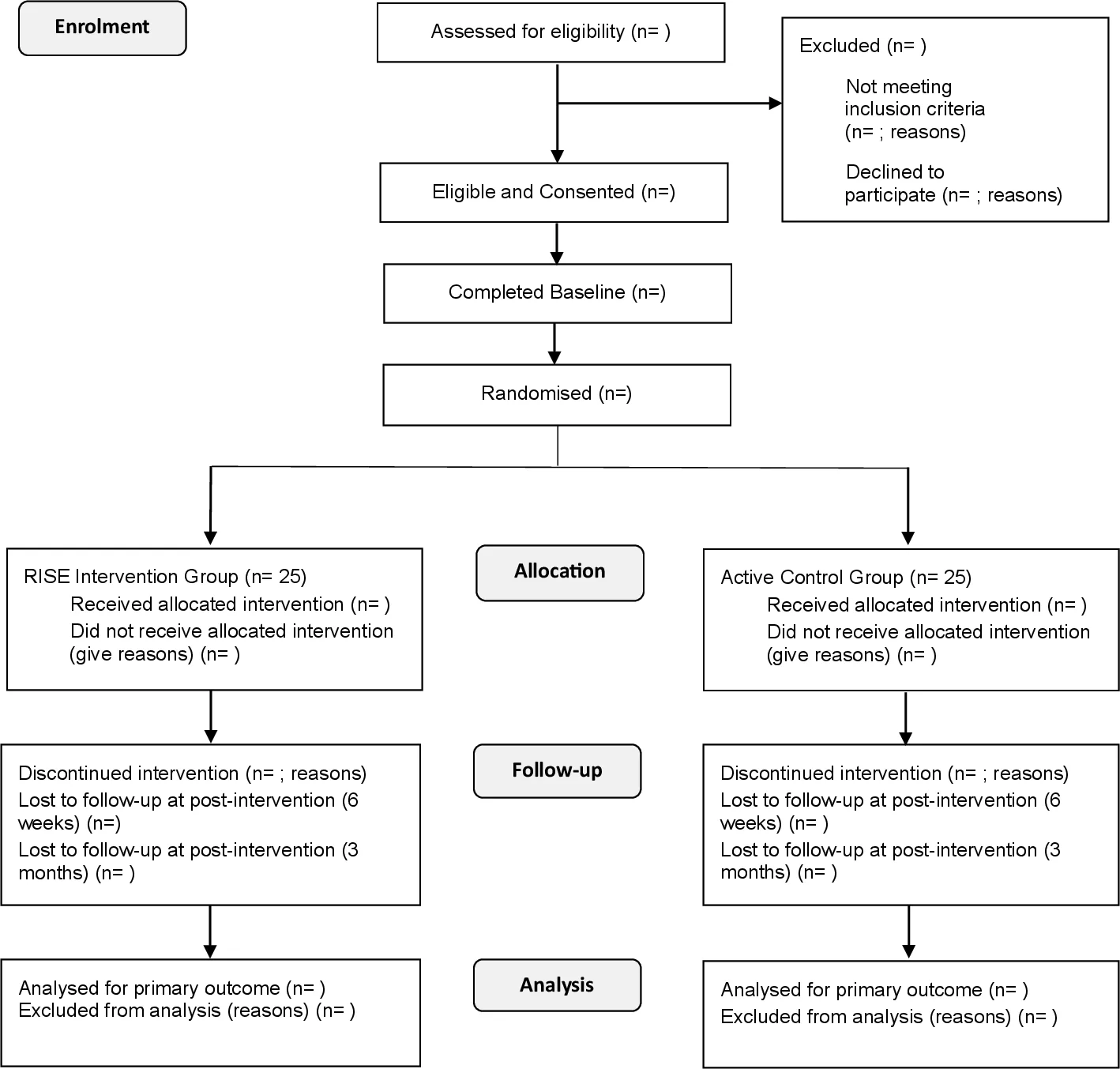
CONSORT 2025 flow diagram. CONSORT, Consolidated Standards of Reporting Trials; RISE, recovery, information, support and empowerment.

Study flow is illustrated in figure 1. This trial will adhere to the CONSORT extension for pilot and feasibility trials^21^ and the Standards for Reporting Implementation Studies (StaRI) Statement and was prospectively registered with the Australian New Zealand Clinical Trials Registry (CTRN12626000506392).

### Participants

Participants will be eligible if they are: (1) aged 18 years or older, (2) diagnosed with WAD grade II/III (no fracture/dislocation) within 12 weeks of RTI, (3) identified at medium or high risk of poor recovery on the WhipPredict risk screening tool^22^ and (4) have access to a mobile phone and reasonable reception. Participants using their own Garmin wearable device will also require a compatible smartphone to operate the associated application (Fitrockr app).

Participants will be excluded if they: (1) demonstrate severe acute psychological distress or safety risk (Kessler Psychological Distress Scale (K6) >13^23^ and psychological risk screening) requiring a higher level of mental healthcare than can be safely provided within the trial intervention; (2) have relevant medical co-morbidities (ie, WAD Grade IV, fracture or injuries to other body areas or recent spinal surgery in the past 12 months or a pre-existing chronic pain condition); (3) are unable to complete study procedures in English or (4) do not have mobile phone access.

### Procedure

#### Pre-enrolment

Potential participants recruited from the EDs will be identified by the triage nurse, doctor or staff delegate either in real-time during triage or patient consultation or on retrospective review of weekly ED presentation lists. Patients identified in real-time will be informed of the study, provided the study flyer and asked to provide verbal consent to be contacted by a member of the research team. Patients identified via retrospective review will be initially contacted via phone (PAH, Sunshine Coast) or via email or SMS (virtual ED site VECS) by site staff to seek verbal consent for the research team to contact them. Contact will be attempted up to three times, and if no contact is made, site staff will send the patient a letter inviting them to initiate contact with the research team if they would like to participate. At trial commencement, ED patient lists will be reviewed to identify potential participants who presented to the ED within the previous 12 weeks. Potentially eligible patients will be contacted in order of referral date, from oldest to newest, to maximise the likelihood of enrolment within the 12-week eligibility window. Recruitment will also involve flyer distribution to the general community (eg, through newsletters, clinic waiting rooms), digital marketing (including social media, paid Google Ads and email distribution methods) and participant referrals from other relevant research studies.

#### Screening and informed consent

Interested individuals will be screened for eligibility via telephone and informed about the study. Eligible participants will receive the Participant Information and Consent Form and provide informed consent electronically via REDCap (https://redcap.health.uq.edu.au). Ineligible individuals will be informed of the reason for ineligibility, offered the option to be contacted for future studies and provided with My Whiplash Navigator^24^ information and advised to consult their general practitioner for distress or pain management where required.

#### Baseline assessment, randomisation and onboarding

Participants who consent to participate will be asked to complete a baseline assessment survey via the REDCap system. Participants who complete the baseline survey are then randomised 1:1 into either the treatment (RISE intervention) or active control condition.

#### Overview of intervention period

Participants in both conditions will receive a welcome message 2 days after the baseline survey is completed to confirm enrolment and maintain contact while onboarding procedures are completed. Participants allocated to the RISE intervention will also be contacted by telephone to confirm their preferred messaging times and where applicable to organise the delivery of the wearable device (Garmin or Empatica EmbracePlus; device type dependent on availability) prior to commencement of the intervention. Instructions for setting up and using the device will also be provided. Participants will be instructed to wear the wearable device as much as possible over the entire intervention period, except when charging, as we are interested in both activity and sleep data. Participants in the RISE group will also be advised of the interactive nature of the intervention, including two-way messaging features, and offered optional support phone (audio-only) sessions during the intervention period, including one 30-min telephone session with a psychologist (or provisional psychologist) and one 30-min telephone session with a physiotherapist trained in the delivery of simple stress management techniques. Sessions will be scheduled as required. These onboarding procedures ensure participants are prepared to receive the RISE intervention and wearable devices prior to message delivery.

Seven days after the baseline survey is completed, participants will begin to receive the onboarding text messages relevant to their assigned condition. This timeframe allows for participants to receive the smartwatch. The onboarding message series will be delivered over 2 days to minimise burden and information overload. These messages will inform participants that they can pause or withdraw from the study at any point by texting SNOOZE or STOP to the trial’s dedicated mobile number. The messages will also include information on the research team, on-demand messages, two-way messaging options and emergency contact details. Prior to commencement of daily messages, a message will be sent to confirm that the wearable device has been set up and is working.

Messages will commence on the first Monday following completion of onboarding to standardise intervention delivery cycles. Commencement of the daily messages will be delayed to the following Monday if the wearable device has not yet been received. Participants will then receive the RISE intervention, which includes receiving approximately one text message daily. Participants in the control condition will begin to receive general health information via text messages every 2 days to support participant masking. Participants in the RISE intervention group will receive daily tailored messages, including the option to engage in interactive two-way messaging, respond to check-in messages and use the weekly reflective diary entry feature. If scheduled, participants in the RISE intervention will complete their psychologist and physiotherapist support sessions during the intervention period. Both interventions will be delivered over a 3 week period following completion of onboarding, with the option of an additional 3 days of messaging support for participants in the RISE group.

#### Post-intervention assessments

All participants will be asked to complete an online assessment questionnaire at 6-week post-randomisation and at a 3-month follow-up. The questionnaire is completed online, and participants will be sent a link via email (or SMS) where they can access it. All participants will be remunerated $30 for each questionnaire completed (based on Health Consumer Queensland guidelines).

A summary of the proposed data collection schedule, in accordance with Standard Protocol Items: Recommendations for Interventional Trials (SPIRIT) guidelines, is outlined in table 1.

**Table 1:** Proposed data collection schedule according to SPIRIT guidelines

	Pre-trial		Trial period			
	Week 0		Week 1	Weeks 2–5	Week 6	3 Months
	Eligibility, informed consent	Baseline questionnaire, random allocation	Intervention onboarding	Intervention period	Follow-up 1	Follow-up 2
**Pre-enrolment**						
Screening						
Inclusion/exclusion criteria	X					
WhipPredict	X					
K6 (Kessler Psychological Distress Scale)	X				X	X
Participant information and informed consent	X					
**Baseline assessment and randomisation**						
Baseline questionnaire		X				
Randomisation		X				
Interventions/comparator						
Intervention onboarding			X			
Welcome message			X			
Phone call (RISE only)			X			
Onboarding messages			X			
Wearable device delivery and set-up check (RISE only)			X			
SMS interventions						
RISE intervention						
Tailored daily messages				X		
My Whiplash Navigator access				X		
Weekly check-in and diary entry				X		
Optional psychologist session				X		
Optional physiotherapist session				X		
Wearable use				X		
Optional continuation SMS				X		
Active control				X		
General health messages				X		
My Whiplash Navigator access				X		
**Assessments**						
Expectancy and acceptability						
Credibility/Expectancy Questionnaire		X				
Acceptability (Technology Acceptance Questionnaire and purpose-built items)		X			X	X
Session Rating Scale (RISE only)				X		
Blinding assessment (perceived group allocation)					X	
Pain and disability						
Pain tolerability and comfort		X			X	X
Pain intensity (Brief Pain Inventory)		X			X	X
Neck Disability Index (NDI)		X			X	X
Psychological						
DASS-21		X			X	X
Post-Traumatic Stress Disorder single items*		X			X	X
Primary care PTSD screen		X			X	X
PCL-5 (only if PTSD screening is positive)		X			X	X
Quality of life and well-being						
EQ-5D-5L		X			X	X
Global Impression of Change					X	X
Cost-related						
Health service and medication use		X			X	X
Compensation claim and lawyer involvement		X			X	X
Work/income status		X			X	X

* These items guide the tailoring of the messages.

DASS-21, 21-item Depression Anxiety and Stress Scale; EQ-5D, EuroQol-5 Dimension (health-related quality of life); GIC, Global Impression of Change; PCL-5, PTSD Checklist for DSM-5 ; PTSD, Post-Traumatic Stress Disorder; RISE, recovery, information, support and empowerment; SMS, short message service; SPIRIT, Standard Protocol Items: Recommendations for Interventional Trials.

### Process evaluation

A process evaluation will focus on key implementation outcomes,^25^ including acceptability, feasibility and perceived appropriateness of the RISE intervention. Following completion of the post-assessment questionnaire, a purposive sample of approximately 10 participants from the RISE intervention group will be invited to complete a semi-structured interview exploring their experience of the RISE intervention including barriers and enablers to participation and suggestions for improvement. Sampling will aim to capture variation in recruitment site, level of intervention engagement and participant experience to obtain a diverse range of perspectives on acceptability and implementation. Participants will receive $50 for completing a 60-min interview. In addition, patients, health professionals and funders (compulsory third-party (CTP) insurance representatives) (total approximately n=10) will be purposively sampled and invited to participate in a series of 2–3 focus groups (or individual interviews if required) to explore opportunities to optimise and integrate RISE into healthcare settings and insurance system care pathways. Patient participants will receive $50/hour for participating in focus groups. These sample sizes are pragmatic and appropriate for a feasibility study and are intended to capture a range of experiences and perspectives to inform intervention refinement and implementation planning rather than to achieve thematic saturation. Findings from the process evaluation will be used to contextualise feasibility outcomes, including recruitment, retention and intervention engagement and to inform interpretation of preliminary clinical outcomes.

### RCT sample size

A formal power calculation was not conducted, as this study is designed as a feasibility trial. A target of 25 participants per arm over 12 months is selected to support estimation of key feasibility parameters and to inform planning for a future definitive trial. This sample size is broadly consistent with recommendations for pilot and feasibility trials, which suggest total sample sizes in the range of 24^26^–70 participants.^27^

### Randomisation

Participants will be randomly assigned to the intervention or control condition in a 1:1 ratio using a computer-generated random allocation list. A statistician who is not involved in data collection will generate the allocation sequence. REDCap will then automatically assign consented participants who have completed the baseline assessment in a 1:1 ratio to either the RISE intervention or active control group using a pre-loaded randomisation table. Allocation will be concealed using a secure, centralised randomisation process within REDCap, ensuring that the research team members enrolling participants cannot predict or influence group assignment.

### Blinding and masking

To minimise potential bias in outcome measurement, outcome measures will be collected through REDCap and monitored by a research officer who is blinded to participant allocation. At follow-up, participants will be explicitly instructed not to discuss any details of the study, including their group assignment, with the research officer to protect the integrity of the blinding process.

Participant masking will be supported by providing both groups with SMS contact, without disclosing group allocation. The usual care group (control condition) will receive two text messages a week on general health information related to whiplash and be prompted to access the publicly available *My Whiplash Navigator*. At any stage, patient care and safety will be prioritised over participant blinding and masking procedures.

### Intervention condition: RISE

The RISE intervention is a tailored text message-based intervention to support the recovery journey for patients with acute WAD from an RTI. It is delivered over a 3–4 week period, where messages will be sent to patients targeting two treatment mechanisms identified in prior research: stress symptom management and improved pain coping skills^14^ in addition to recommended exercise and activity. Message content was further developed in line with themes identified from prior work co-designing the intervention (*manuscript under review*). These themes included: 1) What can I expect in terms of RTI symptoms and the recovery process? 2) What can I do to help myself? and 3) What are these unexpected emotions and trauma responses, and are they normal? The timing, intensity and frequency of the messages will be tailored to patient preferences where possible. RISE includes four core domains, which underpin the content of the messages, as outlined:

1. **R**ecovery-focused messages that provide reassurance, encouragement and emotional support.
2. **I**nformational support providing education on whiplash injury and pain and what to expect during recovery.
3. **S**upport for behaviour change, including psychological techniques.
4. **E**mpowering and motivational messages to enhance patient confidence in managing their pain and recovery.

#### Personalisation of the RISE intervention

The RISE intervention incorporates a range of personalisation features within both message content and delivery to support relevance and responsiveness to individual participant needs and preferences over the intervention period. During onboarding, participants nominate their preferred time of message delivery and are offered the option to schedule one 30-min session with a psychologist and one 30-min session with a physiotherapist trained in StressModex (integrated stress inoculation training and exercise)^13^ at any point during the programme. Throughout the intervention, participants can access additional messages on demand via keyword text requests (eg, CALM, ACTIVATE). At the end of week 1, participants receive a prompt to review message frequency and may choose to reduce frequency for a 24-hour period. In addition, participants can temporarily reduce message frequency at any time by texting SNOOZE. Message content is further tailored during weeks 1–3 based on baseline survey responses (eg, sleep disturbance, concentration difficulties), and participants receive two-way SMS check-ins during this period to rate pain intensity, stress and perceived support. Participants are also invited to complete a brief weekly check-in survey and diary entry to reflect on their experiences. At the end of week 3, participants are offered the option to extend messaging for an additional 3 days. These features provide a structured approach to tailoring intervention delivery while maintaining consistency across participants.

### Active control condition (general health text messages plus an online resource, My Whiplash Navigator)

Participants in the active control group will receive general onboarding messages without options for any intervention or message personalisation. On the first Monday following completion of the onboarding period, they will then receive two messages per week containing general health information and education about WAD recovery and recommended management, including activity and exercise, as well as access to a publicly available website, My Whiplash Navigator. They will receive these messages for the same 3-week period as the intervention group but will not have the option for additional days or clinician-based support.

Messages in both conditions will be delivered using a commercial SMS provider (CellCast, https://www.cellcast.com/au), using a dedicated telephone number. All SMS scheduling and storage will occur within The University of Queensland’s research infrastructure.

### Feasibility outcomes and key feasibility criteria

Feasibility outcomes were selected to reflect key trial processes, RISE intervention engagement, acceptability and the feasibility of collecting outcome and cost data, in line with recommendations for pilot and feasibility studies.^28^ Pre-specified progression criteria will be used alongside information gathered from the process evaluation to determine whether a future definitive RCT is warranted. Criteria are based on a traffic-light system, where green indicates that the feasibility target has been met and progression to a definitive trial is supported; amber indicates that modifications to the protocol are required prior to progression and red indicates that progression to a definitive trial is not currently recommended.

Trial feasibility outcomes (eg, recruitment and retention) will be summarised across all randomised participants, whereas intervention-specific feasibility outcomes (eg, uptake, acceptability and wearable use) will be calculated for participants allocated to the RISE intervention only. Uptake of optional clinician support sessions will be assessed by examining the proportion of scheduled sessions that are completed. Because these sessions are intended to provide additional support where needed, overall demand for sessions will be interpreted descriptively rather than used as a progression criterion. The feasibility outcomes and corresponding progression criteria are presented in table 2.

**Table 2:** Feasibility outcomes and criteria for proceeding to a definitive randomised controlled trial (RCT)

Feasibility outcome	Measurement	Green	Amber	Red
Recruitment	% of eligible participants who consent to participate	70%	50–69%	<50%
	Average number of participants recruited per month	≥4 /month	2–3/month	<2/month
	% who consent to use the wearable device (RISE intervention group only)	50%	30–49%	<30%
Retention	% who complete the outcome assessment at 6-weeks and 3-month assessment	70%	50–69%	<50%
	% who complete the cost-related data questions at baseline, 6 weeks and 3 months	50%	30–49%	<30%
RISE uptake	% of intervention participants who respond to ≥3 interactive SMS prompts during the intervention period (RISE intervention group only)^*^	70%	30–69%	<30%
	% of scheduled psychologist scheduled sessions that are attended (RISE intervention group only)	70%	50–69%	<50%
	% of scheduled physiotherapist scheduled sessions that are attended (RISE intervention group only)	70%	50–69%	<50%
Acceptability†	% sample with usefulness and ease of use mean scores>4.0/5‡ (RISE intervention group only)	60%	40–59%	<40%

Note: Green light indicates the feasibility outcome is achieved and that we can proceed to a full definitive randomised controlled trial (RCT); amber light indicates that changes to the proposal are required before proceeding to a full trial; red light indicates that the feasibility outcome is not achieved and proceeding to a full trial is not recommended.

* Interactive engagement is defined as responding to two-way SMS prompts (eg, check-in ratings). Participants receive approximately three interactive prompts per week on average. Engagement with prompts is encouraged but not required.

† Additional acceptability metrics will include qualitative experience data as part of the process evaluation, self-report responses to the purpose-built and validated questionnaires and behavioural indicators of engagement.

‡ Adapted Technology Acceptance Measure; the mean score is calculated as the mean of items within each subscale. Participants are classified as meeting the criterion if both usefulness and ease-of-use mean scores are ≥4.0.

RISE, recovery, information, support and empowerment; SMS, short message service.

#### Cost-related data

The feasibility of collecting direct and indirect cost data will be assessed. Participants will be asked to report their current medication use, including over-the-counter medication (type, dose, frequency) for management of WAD. They will also be asked to retrospectively report their daily medication use and health service use, including medical (including radiology/imaging), allied health and complementary (eg, massage, acupuncture) over the past week. Participants will also be asked to report their pre-injury and current work status, gross yearly salary range and percentage of regular work capacity, insurance claim status, any legal involvement and any finalised claim payout amounts. These data will be used to inform the design of a future within-trial full economic evaluation. The costs of delivering the intervention (eg, SMS messages, clinician time) will also be estimated.

#### Acceptability

An adapted version of the Technology Acceptance Model (TAM)^29^ will be used to assess perceived ease of use and perceived usefulness of the RISE intervention. Each construct is measured using six items rated on a 7-point scale from 1 (‘Strongly disagree’) to 7 (‘Strongly agree’). Mean scores will be calculated for each subscale, and participants will be classified as meeting the acceptability criterion if both subscale mean scores are≥4.0. TAM items will be framed in the future tense at baseline to assess anticipated acceptability and in the past tense at follow-up to assess experienced acceptability following intervention use.^30^

Additional indicators of acceptability will include responses to purpose-built items, including participant intention to use (or continue using) the intervention, willingness to recommend the intervention to others with WAD and Session Rating Scale scores for participants who complete the clinician-guided session/s.^31^ Intervention-specific items assessing perceived informational support, emotional support, encouragement for behaviour change and confidence in managing recovery will also be included. These items were developed to align with the core domains of the RISE intervention. In addition, behavioural indicators of acceptability will be examined and summarised. These include participant engagement with optional or self-initiated features, such as use of the on-demand message function, ‘snooze’ or ‘stop’ functions and weekly diaries, as well as the frequency of use of these features. Participant feedback on message frequency and perceived helpfulness will also be assessed. These behavioural indicators provide additional insight into patterns of participant engagement beyond the core intervention components. Finally, we will examine time participants wear the device, including the extent of valid data derived from the wearable device.

Patient experience and clinician and funder perspectives, including those of CTP insurance representatives, will be explored as part of the process evaluation. Individual semi-structured interviews and focus groups will examine the perceived value and implementation potential of the RISE intervention as well as opportunities for optimisation. Sessions will be co-facilitated by research staff experienced in co-design and qualitative methods, audio-recorded and transcribed verbatim.

#### Assessment of participant blinding

To assess the success of participant blinding, participants will be asked at the post-intervention follow-up which study group they believe they were assigned to (RISE intervention, active control or unsure). Participants will also rate their confidence in this belief on an 11-point scale (0=not at all confident to 10=extremely confident) and will be invited to provide a brief explanation for their response. Responses will be summarised descriptively to assess the extent to which participants were able to correctly identify their allocation. This information will be used to evaluate the feasibility of maintaining participant blinding in a future definitive trial.

### Health outcomes and screening measures

During the baseline survey, participants will be asked a range of demographic and clinical and health-related questions. Demographic questions include age, gender and socioeconomic information (education, employment, marital status, language and English proficiency, postcode). Clinical, health and technology information will include time since the RTI, current diagnoses and digital and SMS literacy.

#### Risk of poor recovery

Participants will complete the WhipPredict as a screening measure only. Participants are asked to indicate how their neck pain has affected their ability to manage in everyday life, such as pain intensity, personal care, lifting, reading, headache, concentration, work, driving, sleep and recreation. Responses are on a scale of 0–5, with each point indicating how much that item affects them in their everyday life. If a participant is over the age of 35, they are asked to complete five more questions relating to hyper-arousal symptoms. Items are rated on a 4-point scale, ranging from ‘not at all or only one time’ to ‘5 or more times a week/almost always’. The total score obtained indicates the level of risk which is used to predict outcomes such as the likelihood of developing moderate/severe disability or experiencing full recovery from acute whiplash injury.^32^ It demonstrates good prognostic accuracy (area under the curve (AUC) ≈0.73–0.80) for identifying individuals at risk of poor recovery.^32^

#### Psychological distress

Non-specific psychological distress will be assessed using the K6.^23^ It is used during screening to determine the presence of acute psychological distress that would preclude involvement in the study and will be repeated at follow-up time points. Items are measured on a 5-point Likert scale, ranging from 1 (‘All of the time’) to 5 (‘None of the time’) and ask participants to indicate how they have been feeling during the past 30 days (eg, how… nervous, hopeless, restless/fidgety, worthless, that everything was an effort).

The Credibility/Expectancy Questionnaire^33^ assessed at baseline will measure how much participants believe that the intervention will help to improve their recovery. The measure is based around two key aspects of *belief:* (1) what one *thinks* will happen and (2) what one *feels* will happen. Participants will answer 2 sets of questions. Set 1 comprises four questions, three of which are scored on a 9-point scale ranging from 1 (‘Not at all logical’) to 9 (‘Very logical’). Questions include, “At this point, how successfully do you think this intervention will be in reducing your [pain/stress] symptoms?” The last question in Set 1 asks, “By the end of the intervention period, how much improvement in your pain/stress symptoms do you think will occur?” and is answered as a percentage, ranging from 0 to 100%. Set 2 comprises two questions about how participants feel about the intervention and its likely success answered on the same 9-point scale.

#### Pain and disability

The experience of pain will be measured at baseline and at all follow-ups using the Brief Pain Inventory.^34^ Participants rate pain severity by reporting their worst, least, average and current pain over the past 24 hours using 11-point numerical rating scales ranging from 0 (‘No pain at all’) to 10 (‘Pain as bad as you can imagine’). Participants report current pain management strategies and the percentage of pain relief obtained from treatments or medications over the past 24 hours.

Pain tolerability and comfort will be measured at baseline and all follow-ups using single items. Participants rate pain intensity ‘over the past 24 hours’ and ‘right now’ on a scale of 0 (‘No pain’) to 10 (‘Pain as bad as you can imagine’) and their pain tolerability ‘over the past 24 hours’ and ‘right now’ on a scale of 0 (‘Completely tolerable’) to 10 (‘Completely intolerable’); these items are adapted from Markman *et al*.^35^

Using a purpose-built measure, participants rate their level of comfort by reporting their comfort at its worst, at its least, on average and right now on a scale of 0 (‘Not comfortable at all/No comfort’) and 10 (‘The most comfortable I have ever been/High comfort’). Participants also report their level of discomfort on a scale of 0 (‘No discomfort at all/No discomfort’) and 10 (‘The most uncomfortable I have ever been/High discomfort’).

Pain-related disability will be measured at baseline and at all follow-ups using the Neck Disability Index.^36^ Participants are asked to indicate how their neck pain has affected their ability to manage in everyday life, with items related to pain intensity, personal care, lifting, reading, headache, concentration, work, driving, sleep and recreation. Participants answer each item on a scale of 0–5, with each point indicating how much that item affects them in their everyday life.

#### Depression, anxiety and stress

Depression, anxiety and stress symptoms will be measured at baseline and at all follow-ups using the 21-item Depression Anxiety and Stress Scale (DASS-21).^37^ Participants will be asked to read each statement and indicate how much the statement has applied to them over the past week, from ‘0 – Did not apply to me’ to ‘3 – Applied to me very much or most of the time.’ Example statements include, ‘I found it hard to wind down’.

#### Post-traumatic stress symptoms (PTSD)

Post-traumatic stress symptoms will be assessed at baseline and at all follow-ups using a two-stage approach. Participants will first complete the Primary Care PTSD Screen for DSM-5 (PC-PTSD-5),^38^ a 5-item screening measure designed to identify probable PTSD symptoms experienced over the past month. Participants respond ‘Yes’ or ‘No’ to items assessing re-experiencing (eg, unwanted memories or nightmares), avoidance of trauma-related thoughts or reminders, hyperarousal (eg, being on guard or easily startled), emotional numbing or detachment and trauma-related guilt or self-blame. Participants scoring ≥3 on the PC-PTSD-5 will subsequently complete the PTSD Checklist for DSM-5 to assess the severity of PTSD symptoms. The PCL-5^39^ is a 20-item self-report measure assessing DSM-5 PTSD symptom clusters over the past month, with higher scores indicating greater symptom severity.

Additionally, three items adapted from Horwitz^40^ will be used to inform real-time tailoring of the SMS messages. Participants will rate the extent to which they currently feel nervous, worried or anxious they are and the extent to which they are re-hashing the circumstances related to the car accident on a 5-point scale (‘Not at all’ to ‘Extremely’). Participants will also report on their current problems with fatigue on an 11-point scale, with 0 being ‘No problem’ and 10 being ‘Severe problem’.

#### Health-related quality of life

Health-related quality of life will be assessed at baseline and all follow-ups using the EQ-5D-5L.^41^ The EQ-5D-5L measures current health status across five domains: mobility, self-care, usual activities, pain/discomfort and anxiety/depression. For each domain, participants select one of five response options indicating severity of problems, ranging from ‘no problems’ to ‘extreme problems’. Summary Health State Values can be computed using the country-specific value sets.^42^ In addition, using the EQ-VAS, participants rate their overall health ‘today’ on a visual-analogue scale ranging from 0 (‘the worst health you can imagine’) to 100 (‘the best health you can imagine’).

#### Global impression of change

Participants’ perceived change in health and functioning will be assessed at 6-week and 3-month follow-up using an adapted Global Impression of Change.^43^ Participants are asked to evaluate how their overall condition and specific functional domains have changed since commencing treatment, relative to pre-treatment. Domains assessed include overall functioning, physical activities, social activities, work-related activities (including household work), mood and pain. Each item is rated on a 7-point Likert scale ranging from ‘Very much improved’ to ‘Very much worse’.

### Physiological data and other study data and metrics

Data collected during the trial, including wearable-derived physiological metrics (eg, activity and sleep), participant-reported outcomes, SMS engagement metrics (eg, response patterns, use of on-demand keywords, message ratings) and qualitative feedback from the diary entries will be securely stored for subsequent integrated analysis. These data will be used in an exploratory phase to examine how multimodal data sources can be combined to inform future optimisation of message content, tailoring and intervention delivery. Participants will receive an additional $30 per week for using the wearable device and completing the check-ins (paid once at the end of the intervention).

### Data analysis

Participant characteristics and baseline measures will be summarised using descriptive statistics. As this is a feasibility trial, analyses will be primarily descriptive and will focus on estimating feasibility parameters. Feasibility outcomes (recruitment, retention, intervention engagement, data completeness and acceptability) will be descriptively summarised. These estimates will be interpreted against the pre-defined progression criteria (see table 2) together with the process evaluation data to determine whether a future definitive trial is warranted. Final progression decisions will be made by the Trial Management Committee based on the overall pattern of feasibility findings across domains, together with qualitative process data and consideration of whether any identified barriers are modifiable. Where findings are mixed, decisions regarding progression will consider the pattern of results across domains, the nature and modifiability of barriers and the potential for refinements prior to a future trial. Participant responses regarding treatment allocation will also be summarised descriptively and compared with actual allocation to assess the success of participant blinding. No formal imputation of missing data is planned. The extent and patterns of missing data will be examined as part of the feasibility evaluation of the data collection procedures.

Preliminary changes in patient-reported outcomes will be explored using estimates of between-group differences with 95% CIs at each follow-up time point. These analyses will be conducted on an intention-to-treat basis. As this feasibility study is not powered to evaluate clinical effectiveness, no formal hypothesis testing will be undertaken and estimates will be interpreted cautiously with consideration of the width of the CIs. These exploratory analyses are intended to identify preliminary signals of potential clinical benefit, assess the suitability and responsiveness of candidate outcomes and inform the selection of the primary clinical outcome for a future definitive trial.

Qualitative interview and focus group data will be analysed using reflexive thematic analysis^44 45^ to explore participant acceptability of the RISE intervention, barriers and enablers to implementation and opportunities for intervention optimisation and integration into health and insurance systems’ models of care. This will involve repeated reading of the transcripts to achieve immersion in the dataset and to note initial analytic observations. Data will then be systematically coded to capture meaningful features relevant to the research question, and codes will be collated with their associated data extracts. Related codes will be grouped to generate preliminary themes, which will be reviewed and refined through comparison with both the coded data and the full dataset to ensure they provide a coherent and credible account. Themes will then be clearly defined, named and developed into a detailed analytic narrative, supported by illustrative data extracts and interpreted in relation to the existing literature. Analysis will be iterative and will inform refinement of the intervention and the design and implementation strategy for a future definitive trial. It will be conducted by research staff with experience in qualitative analysis.

Self-reported medication use, health service utilisation and income/productivity impacts (eg, time off work) will be collected and converted into estimated costs using standard Australian unit cost sources (eg, PBS and MBS schedules and national average wage estimates where applicable). Costs will be calculated per participant over the reporting period. This approach is intended to assess the feasibility of collecting and costing resource use and productivity data, rather than to generate precise cost estimates.

An exploratory analysis will be undertaken using a large language model (LLM; Microsoft Copilot) to integrate and interpret multimodal data. This analysis is developmental in nature and will be used to explore how data sources may be integrated to inform future optimisation of message content, tailoring and intervention delivery. All AI-supported analyses will be conducted on de-identified data in accordance with institutional data governance and ethical approvals.

### Trial oversight

The RISE Trial Management Committee, comprising the chief investigator, members of the research team with methodological and clinical expertise, hospital site investigators and consumer representation, will oversee the conduct and progress of the study, as a Data Safety Monitoring Board is not warranted due to relatively low risks associated with this trial.^42^ The committee will oversee study implementation in accordance with the approved protocol, review progress against study objectives and advise on any protocol or operational issues arising during the feasibility trial. The committee will meet at regular intervals and more frequently if required to fulfil its oversight functions. The chief investigator will be responsible for the day-to-day management of the study, including coordination of research activities across sites.

### Patient and public involvement

People with lived experience of RTI and WAD were involved in the co-design of the RISE intervention and contributed to the development of study procedures, participant materials and outcome selection. Consumer representation on the RISE Trial Management Committee will provide ongoing input into study conduct and interpretation of findings, and two consumer representatives have been included as co-authors on this study. Participant feedback collected during the feasibility trial will inform intervention refinement and the design of a future definitive trial. Study findings will be disseminated in partnership with consumer representatives.

## Discussion

This study will evaluate the feasibility and acceptability of delivering RISE, a co-designed, clinician-supported SMS-based early intervention for individuals with WAD in the first 12 weeks following a RTI. By integrating psychological strategies with recommended exercise in a low-intensity, scalable digital format, the trial addresses a critical gap between guideline recommendations and access to early targeted intervention for those at risk of poor recovery. The mixed-methods design will provide comprehensive insight into recruitment and retention processes, intervention engagement, outcome measure feasibility and participant experience, while also exploring implementation considerations within clinical and insurance care pathways. The inclusion of wearable-derived physiological data and exploratory large language model-supported data integration represents an innovative approach to informing future personalisation and optimisation of digital health interventions in injury populations. Several personalisation features identified during the intervention co-design are incorporated to allow adaptability of the intervention, including the opportunity for participants to access brief clinician support from both a psychologist and physiotherapist. The level of interest in and uptake of these features will provide valuable insight into patient preferences and inform future refinement of the intervention.

Findings from this study will determine whether progression to a definitive trial is warranted and will inform its design, including intervention refinement, outcome selection and implementation strategies to support translation into routine care. Given the high prevalence and burden of WAD and the recognised challenges in delivering timely, targeted early care, scalable digital interventions may offer a practical strategy to bridge gaps between guideline recommendations and real-world practice. If feasible, RISE has the potential to complement existing clinical care pathways by providing accessible early psychological and behavioural intervention, with opportunities for personalisation and integration within healthcare and insurance systems.

### Ethics and dissemination

Ethical approval for this study has been obtained from the Townsville Hospital and Health Service Human Research Ethics Committee (HREC/QTHS/121395) and ratified by The University of Queensland Human Research Ethics Committee (2025/HE002491). The study will be conducted in accordance with the National Health and Medical Research Council National Statement on Ethical Conduct in Human Research (2007, updated 2018) and the principles of the Declaration of Helsinki.

All participants will provide informed consent prior to participation (see online supplemental material for a copy of the participate information and consent form). For participants recruited via hospital services, a member of the treating team or research staff will introduce the study with verbal consent obtained prior to sharing participant contact details for the research team to follow-up. Participants can also contact the research team directly if they receive a letter or see study advertisements. Participation is voluntary and participants may withdraw at any time without consequence to their care.

Given the nature of the co-designed intervention, risks to participants are considered low. The RISE intervention consists of supportive and informational text messages designed to promote recovery. Participants will be able to opt out of receiving messages at any time (eg, by replying ‘STOP’) or temporarily pause messages if desired. While the content is not intended to replace clinical care, participants will be encouraged to seek appropriate medical advice if symptoms worsen or concerns arise. Any adverse events or participant concerns raised during the trial will be monitored and managed in accordance with study protocols.

Participant confidentiality will be maintained throughout the study. Data will be de-identified and stored securely on password-protected servers at The University of Queensland. Only authorised members of the research team will have access to identifiable data. Any data used for analysis and dissemination will be reported in aggregate form to ensure participants cannot be identified.

Findings from this feasibility trial will be disseminated through peer-reviewed publications, conference presentations and stakeholder reports. Results will also be shared with participating clinical sites and relevant stakeholders, including clinicians, researchers and policy-makers. Where appropriate, summaries of findings will be provided in plain language to participants and consumer representatives involved in the co-design process to support transparency and knowledge translation.

## Supplementary material

10.1136/bmjopen-2026-123039 online supplemental file 1

## Data Availability

Data sharing not applicable as no datasets generated and/or analysed for this study.
