# Supplementary material for: RISE for recovery after acute whiplash injury: protocol for a randomised feasibility trial of a clinician-supported SMS-based behavioural intervention
Source: BMJ Open. 2026 Sep 11;16(9):e123039. doi: 10.1136/bmjopen-2026-123039 (PMC13583748; doi:10.1136/bmjopen-2026-123039)
Supplement: Supplementary file 1 [file bmjopen-2026-123039.reviewer_comments.pdf]

# PEER REVIEW HISTORY

BMJ Open publishes all reviews undertaken for accepted manuscripts. Reviewers are asked to complete a checklist review form and are provided with free text boxes to elaborate on their assessment. These free text comments are reproduced below.

## ARTICLE DETAILS

**Title (Provisional)**

RISE for Recovery after Acute Whiplash Injury: Protocol for a Randomised Feasibility Trial of a Clinician-Supported SMS-based Behavioural Intervention

**Authors**

Elphinston, Rachel A.; Formosa, Jessica; Armfield, Nigel; van der Vegt , Anton H; Ashton-James, Claire E; Brain, Katherine; Buckley, Lisa; Papic, Christopher; Khan , Asaduzzaman; Eather, Chloe-Emily; Liimatainen, Jenna; Hobbins King, Andrew; Hansen, Kim; Staib, Andrew; Smith, Gaylene M; Chen, Tina; Wright, Karen; Garrad, Elizabeth; Sterling, Michele

---

## VERSION 1 - REVIEW

---

| | |
|---|---|
| **Reviewer** | **1** |
| **Name** | **Williams, Mark** |
| **Affiliation** | **Oxford Brookes University, Sport, Health Sciences and Social Work** |
| **Date** | **16-Jun-2026** |
| **COI** | **None** |

---

The authors are to be commended on a thoroughly well designed and reported study protocol addressing an important question in managing acute Whiplash Associated Disorders via a randomised controlled feasibility trial with embedded mixed methods process evaluation.

The justification for the study is clearly articulated and well evidenced.

The methods are transparently reported so the study conduct can be evaluated and replicated in terms of recruitment, selection, randomisation & allocation concealement, interventions, data collection, analysis of feasibility and acceptability.

Ethical approval and trial governance and registration in place.

A very small number of minor queries:

1. For the study aim - (abstract p.3 and intro p.7) could/should the phrasing be expanded to be ..."to evaluate the feasibility of delivering and evaluating effectiveness of RISE..."? Given

some of the primary feasibility criteria pertain to % completion of outcome assessment" (hypothetically you can have a scenario where it is feasible to deliver (green light on recruitment and RISE uptake) but not on retention.

2. p.11 Screening and informed consent. Can you clarify how potential participants get any questions answered before consenting?

3. tense typo line 8 p.12 - change "were" to "are".

4. p.27 Data analysis - more of a question than a revision request - it is unclear why it is necessary to report between-group differences of changes to PROMS given you will not be hypothesis testing and are not powered for this. Sample size calculation parameters could be gained without looking at between-group differences.

Thank you

---

## VERSION 1 - AUTHOR RESPONSE

Response to Reviewer

Thank you to the Reviewer for the kind comments. We have addressed the minor queries below and have noted additional minor updates we have made to the paper.

A very small number of minor queries:

1. For the study aim - (abstract p.3 and intro p.7) could/should the phrasing be expanded to be ..."to evaluate the feasibility of delivering and evaluating effectiveness of RISE..."? Given some of the primary feasibility criteria pertain to % completion of outcome assessment" (hypothetically you can have a scenario where it is feasible to deliver (green light on recruitment and RISE uptake) but not on retention.

Thank you for this suggestion. We have made these changes accordingly.

2. p.11 Screening and informed consent. Can you clarify how potential participants get any questions answered before consenting?

Screening will be conducted over the phone and the patient will have a chance to ask any questions at this time. I have clarified this on pg. 11

“Interested individuals will be screened for eligibility via telephone and informed about the study”.

3. Tense typo line 8 p.12 - change "were" to "are".

This has been corrected.

4. p.27 Data analysis - more of a question than a revision request - it is unclear why it is necessary to report between-group differences of changes to PROMS given you will not be hypothesis testing and are not powered for this. Sample size calculation parameters could be gained without looking at between-group differences.

Thank you for these comments. We have made some edits to this section to clearly articulate the preliminary nature of these and the purpose (pg. 28).

“Preliminary changes on patient-reported outcomes will be explored using estimates of between-group differences with 95% confidence intervals at each follow-up time point. These analyses will be conducted on an intention-to-treat basis. As this feasibility study is not powered to evaluate clinical effectiveness, no formal hypothesis testing will be undertaken, and estimates will be interpreted cautiously with consideration of the width of the confidence intervals. These exploratory analyses are intended to identify preliminary signals of potential clinical benefit, assess the suitability and responsiveness of candidate outcomes, and inform selection of the primary clinical outcome for a future definitive trial.”

---
