# Supplementary material for: RISE for recovery after acute whiplash injury: protocol for a randomised feasibility trial of a clinician-supported SMS-based behavioural intervention
Source: BMJ Open. 2026 Sep 11;16(9):e123039. doi: 10.1136/bmjopen-2026-123039 (PMC13583748; doi:10.1136/bmjopen-2026-123039)
Supplement: Supplementary file 2 [file bmjopen-16-9-s001.docx]

**RECOVER Injury Research Centre**

**Participant Information Sheet/Consent Form**

**Consumers**

**Version 4 17.07.2026**

| **Title** | Digital intervention after whiplash injury |
| --- | --- |
| **Short Title** | Digital intervention after injury |
| **Protocol Number** | 1 |
| **Project Sponsor** | University of Queensland |
| **Coordinating Principal Investigator/ Principal Investigator** | Dr Rachel Elphinston, PhD – RECOVER Injury Research Centre, The University of Queensland |
| **Associate Investigator(s)** | Prof Michele Sterling, PhD – RECOVER Injury Research Centre, The University of Queensland  Dr Nigel Armfield, PhD - RECOVER Injury Research Centre, The University of Queensland  A/Prof Clair Ashton-James, PhD – Pain Management Research Institute, University of Sydney  A/Prof Lisa Buckley, PhD – MAIC/UniSC Road Safety Research Collaboration, University of the Sunshine Coast  Dr Christopher Papic, PhD - RECOVER Injury Research Centre, The University of Queensland  Dr Anton Van Der Vegt, PhD – Centre for Health Services Research, The University of Queensland  Dr Katherine Brain, PhD – Chronic Brain Australia, University of Newcastle  Dr Kim Hansen, MBBS – Virtual Emergency Care Services, Metro North  Dr Andrew Staib, MBBS – Princess Alexandra Hospital, Metro South  Dr Andrew Hobbins King, BSc MBChB FACEM DCH – Sunshine Coast University Hospital, Sunshine Coast Hospital and Health Service  Bryce McMahon – RECOVER Injury Research Centre, The University of Queensland  Jenna Liimatainen, BExPhys(Hons) -- RECOVER Injury Research Centre, The University of Queensland  Chloe-Emily Eather, BPsySci(Hons) – RECOVER Injury Research Centre, The University of Queensland |
| **Location** | The Virtual Emergency Care Service  Princess Alexandra Hospital  Sunshine Coast University Hospital |

**Part 1	What does my participation involve?**

**1	Introduction**

You are invited to take part in this research project to test a new digital intervention to support recovery early after a road traffic injury. You have been invited because you have recently been diagnosed with a whiplash associated disorder (in the last 12 weeks).

This Participant Information Sheet/Consent Form tells you about the research project. It explains the intervention involved and the processes involved with taking part. Knowing what is involved will help you decide if you want to take part in the research.

Please read this information carefully. Ask questions about anything that you don’t understand or want to know more about. Before deciding whether or not to take part, you might want to talk about it with a relative, friend or your local doctor.

Participation in this research is voluntary. If you don’t wish to take part, you don’t have to. You will receive the best possible care whether or not you take part.

If you decide you want to take part in the research project, you will be asked to sign the consent section. By signing it you are telling us that you:

• Understand what you have read

• Consent to take part in the research project

• Consent to have the tests and treatments that are described

• Consent to the use of your personal and health information as described.

You will be given a copy of this Participant Information and Consent Form to keep.

**2	What is the purpose of this research?**

Providing early and effective treatment of whiplash associated disorders is important and can help to improve recovery. Using digital health technologies can provide easy access to support. In this project, we will test how education and support can be provided using text messages for patients who have experienced a whiplash injury in the last 12 weeks.

Our team has worked together patients and clinicians to develop the text messages to ensure they are supportive and helpful.

This research is being conducted by the RECOVER Injury Research Centre, which is a joint initiative of the Queensland Motor Accident Insurance Commission (MAIC) and the University of Queensland. The RECOVER Injury Research Centre is also a member of the STARS Education and Research Alliance.

**3	What does participation in this research involve?**

**Participant Eligibility**

Participants for this study must be aged 18 years or older, be diagnosed with a whiplash associated disorder from a road traffic injury in the last 12 weeks, and report symptoms that suggest they may be at risk of poor recovery. Participants must be able to speak English and have access to a mobile phone and reasonable reception (so the text messages can be received and responded to).

Individuals experiencing acute distress, and who have acute medical co-morbidity may not be eligible to participate in this study.

If eligible, you will complete online surveys before and after the digital intervention and at 3-months follow-up. The surveys will take approximately 20-30 minutes to complete.

You will be allocated to one of two groups. Both groups will receive education and support via text messages, with some differences in the way and timing the information is provided. To try to make sure the groups are the same, each participant is put into a group by chance (random).

You will then receive the digital intervention via text messages over a period of 3 to 4 weeks and follow the instructions provided in the messages.

Some people may also be invited to use a wearable device during the study. For those who are invited to do so, we will organise with you a suitable collection or delivery method. We have provided details on the wearable device options below in sections 4a and 4b.

At the end of the project, you may be invited to an interview to share your experiences of participating in the study.

This will be a blind study. In a blind study you do not know which of the treatments you are receiving. The research team will know which treatment you are receiving. This research project has been designed to make sure the researchers interpret the results in a fair and appropriate way and avoids the research team members or participants jumping to conclusions.

**AI Use for Research Purposes**

Please note that participant data may be used in an external AI tool (Co-pilot) for research purposes. Specifically, at the end of the trial period, physiological data from the smartwatches may be analysed using a large language model (LLM), such as Co-pilot, in conjunction with study data to help inform AI use in the intervention. Data collected during the study may also be used in the LLM to refine and further develop text messages for the RISE intervention.

**Time Commitment**

In total, your involvement in this study will take approximately 4-6 weeks to complete, with a follow up questionnaire to be completed three months later.

Specifically, participation will involve completing a baseline questionnaire after providing consent, which will take approximately 20-30 minutes. You will then be randomly allocated to one of two groups. It is expected that this process will only take a couple of days to complete. Once allocated to your group, you will receive the onboarding text messages, which provide key information on the study and text messages.

The first Monday after receiving the onboarding messages, participants will begin to receive the text messages as part of the intervention phase. This will last approximately 3-4 weeks.

At the end of the 3–4-week intervention, participants will be asked to complete another questionnaire which will take approximately 20-30 minutes. At this point, participants may also be asked to complete an interview via phone or online, which may take 30-45 minutes to complete.

Three months after the initial baseline questionnaire, participants will be contacted again to complete another 20–30-minute questionnaire. See below for an itemised list of key time commitments:

**Week 1**

•Eligibility screening interview [5-10 minutes]

•Informed consent and completion of baseline questionnaire [20-30 minutes]

•Randomly allocated to group

•Receive onboarding text messages [2-7 days]

**Week 2**

•First text message of the intervention period is sent on the first Monday after onboarding

**Week 2-5**

•Intervention period – receiving regular text messages over 3-4 weeks

**Weeks 5-6**

•Complete follow-up questionnaire [20-30 minutes]

•Online interview scheduled at suitable time if invited and consented [30-45 minutes]

**3 Months after Baseline Questionnaire**

•Complete follow-up questionnaire [20-30 minutes]

**4	What do I have to do?**

*Before the Intervention*

The first steps of the study will involve all participants providing informed consent, completing a baseline questionnaire online, and being randomly allocated to one of two treatments.

The baseline questionnaire will include a variety of measures, including demographic characteristics, pain intensity and interference, mental health, quality of life, and wellbeing. It is estimated that it will take approximately 20-30 minutes to complete.

*During the Intervention*

Participants will receive onboarding text messages to introduce them to the project. The first Monday following onboarding, participants will begin to receive messages as part of the intervention. This will last for approximately 3 to 4 weeks.

Participants may also be asked to track their health and physiological parameters via a wearable device (Empatica EmbracePlus or Garmin vívosmart 5) during this time.

Collection of the wearable device will be organised with each participant, with the option to have the device sent directly to the participant or the option to have the participant collect the device from the RECOVER Injury Research Centre and the Surgical Treatment and Rehabilitation (STARS) Hospital. Using the instructions provided, participants will set up the device and wear it on their wrist. Participants will also be asked to download the associated app to their smartphone (either the Care Lab or Fitrockr App). The research team can be contacted regarding any questions or concerns related to the wearable device and set up. At the end of the intervention, participants will be contacted with instructions on how to return the device, with options including mailing the device back to the RECOVER Injury Research Centre or returning it directly to the centre.

*After the Intervention*

Participants will complete an online questionnaire at approximately 3-4 weeks after the initial baseline questionnaire, once the intervention has been completed. This questionnaire will once again involve questions related to pain, mental health, quality of life, and wellbeing. Participants will also complete this questionnaire at 3 months after baseline questionnaires.

Participants may be asked to participate in an interview at the end of the study, approximately 4 to 6 weeks after the initial baseline questionnaire to share experiences of participating in the project. This interview will be conducted online via Zoom.

Participant involvement in the intervention is estimated to be 6 weeks, with a final online questionnaire completed at three months follow-up.

**4a**	**How are the wearable devices involved in this research project? What do you need to do?**

As part of this research project, you may be asked to track your health and physiological data using one of two watches, depending on availability: the Empatica EmbracePlus smartwatch or the Garmin vívosmart 5 smartwatch.

These wearable smartwatches are simple in design to ensure all-day comfort.

The sizing of the Empatica EmbracePlus smartwatch ranges from 110mm-215mm in wrist circumference, and from 18mm-21mm in width, depending on the watch band provided. The sizing of the Garmin vívosmart 5 smartwatch is approximately 19.5 x 10.7 x 255 mm and fits wrists with a circumference of 148-228 mm. It weighs approximately 26.5g. The below images are an example of what the smartwatches may look like:

**Source:** [**https://www.empatica.com/en-int/embraceplus/**](https://www.empatica.com/en-int/embraceplus/) **Source:** [**https://www.garmin.com/en-AU/p/782585/**](https://www.garmin.com/en-AU/p/782585/)

If you are asked to use one of these smartwatches, you will receive a detailed instruction guide on how to wear and use it, along with its associated app. The Empatica EmbracePlus uses the Care Lab app. The Garmin vívosmart 5 uses the Fitrockr app.

The associated app will need to be downloaded and installed on your smartphone. The research team will provide you with detailed information on how to do this. You will also be able to contact the research team for further assistance if needed (see contact details in section 20).

During the study, if asked to wear a smartwatch, you will need to interact with the associated app regularly to ensure it is working correctly and that the battery is charged. Your health data can be checked in the app.

Please note, we are unable to collect health and physiological data from other wearable devices. If you currently use a different smartwatch, we recommend temporarily removing it and wearing the provided study watch for the duration of the message intervention (approximately 3-4 weeks). However, this is entirely your choice. Additionally, if you’re asked to wear the Garmin vívosmart 5 and already own and wear a Garmin watch, the associated Garmin Connect App will need to be uninstalled from your phone for the duration of the intervention and replaced with the Fitrockr App (approx. 3-4 weeks). This is because the Fitrockr App will not be able to collect all the necessary data needed if the Garmin Connect App is also on installed on your phone.

The Care Lab app is available to be downloaded on suitable smartphones (information on device compatibility can be found here <https://www.empatica.com/embraceplus-compatibility/>). Further information on the EmbracePlus can be found on the Empatica website <https://www.empatica.com/en-int/embraceplus/>.”

Further information on the Garmin vívosmart® 5 watch can be found here <https://www.garmin.com/en-AU/p/782585/>. Further information on Fitrockr can be found here <https://www.fitrockr.com/>

**4b**	**How is data collected via the wearable device?**

If asked to wear a smartwatch for this study, it will track a number of different physiological outcomes, such as steps and activity, sleep, energy expenditure, respiratory rate, and heart rate variability.

The data collection and management process for each smartwatch is outlined below.

**Empatica EmbracePlus**

Data collection and management of the physiological parameters will happen through the Empatica Health Monitoring Platform, which is a modular, cloud-based remote health monitoring platform (https://www.empatica.com/en-int/platform/home).

The process of data collection and management involves the EmbracePlus smartwatch collecting data and transferring it to the Care Lab App via Bluetooth.

Collected data is then moved from the Care Lab App through the smartphone’s internet connection to the Empatica Cloud (<https://www.empatica.com/en-int/cloud-api/>), which securely stores the data and makes it available to the research team via a direct download or through online visualisation on the platform’s Care Portal (<https://www.empatica.com/en-int/care>). The Care Portal allows the research team to then access and manage the data.

Please note that the Empatica Cloud infrastructure is located in Virginia, USA. You can find more information on Empatica’s privacy policy, including the storage of user data on Empatica’s User Privacy Policy here <https://www.empatica.com/en-int/privacy-participant/>.

**Garmin vívosmart 5**

Data collection and management of the physiological parameters will happen through the Fitrockr Health Data Research & Healthcare Platform, which is a cloud-based remote health monitoring platform (<https://www.fitrockr.com/research/>).

The process of data collection and management involves the Garmin vívosmart 5 watch collecting data and transferring it to the Fitrockr App which will be installed on your smartphone.

Collected data is then synced to the Fitrockr Health Data Research & Healthcare Platform through the Fitrockr App, which securely stores the data and makes it available to the research team. Fitrockr is based in and hosts its data in Germany. <https://www.fitrockr.com/research/#howitworks>

**5**	**Other relevant information about the research project**

In total, this study will involve approximately 50 participants. Participants will be recruited from various sources, including hospital emergency departments, virtual emergency departments, as well as from the community.

**6	Do I have to take part in this research project?**

Participation in any research project is voluntary. If you do not wish to take part, you do not have to. If you decide to take part and later change your mind, you are free to withdraw from the project at any stage.

If you do decide to take part, you will be given this Participant Information and Consent Form to sign and you will be given a copy to keep.

Your decision whether to take part or not to take part, or to take part and then withdraw, will not affect your routine treatment, your relationship with those treating you or your relationship with The University of Queensland or Townsville Hospital and Health Services.

**7	What are the possible benefits of taking part?**

We cannot guarantee or promise that you will receive any benefits from this research. However, we hope that you will find your participation and contribution to the study valuable. Participants may benefit from the information provided in the text messages they receive.

Reimbursement

All participants will be reimbursed for their time with $30 for each questionnaire completed (total of $90), plus $30 per week for three weeks as reimbursement for wearing the smartwatch (total $90, paid at the end of the intervention period), as guided by the Health Consumer QLD guidelines. Participants who complete an interview at the end of the study will also be reimbursed $50 for their time.

Overall, the findings of the study will help us develop more effective approaches to the management of whiplash associated disorders. A summary of the findings of this study will be prepared once the information collected has been completed. You can request to receive a copy of the findings of the study by contacting Dr Rachel Elphinston via email (rachelel@uq.edu.au).

**8	What are the possible risks and disadvantages of taking part?**

Some people who have experienced a whiplash injury may experience feelings of stress during the early stages of recovery. Any feelings of stress are likely to be temporary and manageable.

To ensure participants’ psychological safety during this study, important information and contact details on who you can contact if you need further support or help will be provided during the onboarding process and at various times during the intervention. Participants can contact Dr Rachel Elphinston, a clinical psychologist ([rachelel@uq.edu.au](mailto:rachelel@uq.edu.au) or 0412 662 084) if they have any questions about their psychological well-being or would like assistance in accessing the right support.

Participants will also have the option to cease their participation if they wish at any time.

**9**	**What if I withdraw from this research project?**

If you decide to withdraw from this project, you may do so at any time. If you do decide to withdraw from this project, please notify a member of the research team before you withdraw. If you do withdraw, you will be asked to complete and sign a ‘Withdrawal of Consent’ form; this will be provided to you by the research team.

If you decide to leave this research project, the researchers will not collect any additional information from you, although information already collected will be retained to ensure that the results of the research project can be analysed properly and to comply with law. You should be aware that data collected up to the time you withdraw will form part of the research project results. If you do not want your data to be included, you must inform the researchers when you withdraw from the research project.

**10	Could this research project be stopped unexpectedly?**

This research project may be stopped unexpectedly for a variety of reasons, though this is very unlikely to happen. These may include reasons such as:

• Unintended adverse events

• The intervention being shown not to be effective

• Decisions made in the commercial interests of the sponsor or by local regulatory/health authorities.

**11	What happens when the research project ends?**

Upon completion of this research project, access to the SMS text message intervention will no longer be available.

If you would like to receive a summary of the findings of this study, you are welcome to request this by contacting Dr Rachel Elphinston via email ([rachelel@uq.edu.au](mailto:rachelel@uq.edu.au)). A summary of the findings will become available once the information collected for this project has been completed.

**Part 2	How is the research project being conducted?**

**12	What will happen to information about me?**

**Privacy and Confidentiality**

By signing the consent form, you consent to the research team and relevant research staff collecting and using personal information about you for the research project.

The personal information that the research team collects and uses includes socio-demographic information (e.g., age, sex, mobile phone number) and basic clinical details (e.g., injury details). Participants may provide their mailing address to the research team in order to have the wearable device delivered to them, however this personal information, along with email addresses, is collected and stored separately from all other data collected.

Due to the nature of the study being a text message-based digital intervention, the collection of mobile phone numbers is required and will be used in order to send and receive text messages. All text processing and storage occurs within UQ’s research infrastructure; text messages (both sent and received) will be briefly held on CellCast’s infrastructure (<https://www.cellcast.com/au>), which is the text message Service Provider used to send and receive the text messages for the feasibility trial, while in transit to/from participants. All information on CellCast’s data security can be found here <https://www.cellcast.com/au/legal/data-security> and their privacy policy here <https://www.cellcast.com/au/legal/privacy-policy>.

Any information obtained in connection with this research project that can identify you will remain confidential. Information gathered will be stored in a re-identifiable form, meaning that personal details (e.g., name) will be kept separate from the research data, which will be labelled using a study ID code. The list of codes and corresponding participant names (and other personal information) will be kept separate to coded data in a password protected format.

All electronic data will be stored on a UQ RECOVER Injury Research Centre computer, only accessible to the researchers via password protection on the UQ Research Data Manager server (dedicated, secure network drive for storage of research data). Paper data (e.g., consent forms, workshop notes) will be stored in a locked cabinet at RECOVER Injury Research Centre accessible only by the research team.

Any data used to refine and further develop the intervention using AI large language models such as Co-pilot will be completely non-identifiable (that is, there is no way anyone will be able to identify any details about you, or the information you provide). This information will be used only for research and education purposes.

Following completion of the study, the data will be securely archived with password protection on the UQ Research Data Manager server. Data will be stored according to the University's Research Data and Materials Management Policy- 4.20.06. After a minimum of 7 years, data will be disposed of by deleting electronic files and/or shredding paper records.

Your information will only be used for the purpose of this research project and it will only be disclosed with your permission, except as required by law.

**Dissemination of Results**

It is anticipated that the results of this research project will be published and/or presented in a variety of forums. In any publication and/or presentation, information will be provided in such a way that you cannot be identified, except with your permission. To ensure that your personal information cannot be identified, participant ID codes will be used and any potentially identifiable information will be anonymised.

Information about your participation in this research project may be recorded in your health records.

Any information obtained for the purpose of this research project that can identify you will be treated as confidential and securely stored. It will be disclosed only with your permission, or as required by law.

**13**	**Complaints and compensation**

If you suffer any injuries or complications as a result of this research project, you should contact the study team as soon as possible and you will be assisted with arranging appropriate medical treatment. If you are eligible for Medicare, you can receive any medical treatment required to treat the injury or complication, free of charge, as a public patient in any Australian public hospital.

**14	Who is organising and funding the research?**

This research project is being led by Dr Rachel Elphinston from The University of Queensland.

This research has been funded by the MIRAA MAIC grant.

No member of the research team will receive a personal financial benefit from your involvement in this research project (other than their ordinary wages).

**15**	**Who has reviewed the research project?**

All research in Australia involving humans is reviewed by an independent group of people called a Human Research Ethics Committee (HREC). The ethical aspects of this research project have been approved by the Townsville Hospital and Health Service HREC..

This project will be carried out according to the *National Statement on Ethical Conduct in Human Research (2025)*. This statement has been developed to protect the interests of people who agree to participate in human research studies.

**16	Further information and who to contact**

If you want any further information concerning this project or if you have any problems which may be related to your involvement in the project, you can contact the research team by emailing either Dr Rachel Elphinston ([rachelel@uq.edu.au](mailto:rachelel@uq.edu.au)) or Bryce McMahon

**Contact persons**

| Name | Dr Rachel Elphinston |
| --- | --- |
| Position | Senior Research Fellow |
| Telephone | 0412 662 084 |
| Email | [rachelel@uq.edu.au](mailto:rachelel@uq.edu.au) |

| Name | Bryce McMahon |
| --- | --- |
| Position | Senior Research Coordinator |
| Email |  |

For matters relating to research at the site at which you are participating, the details of the local site complaints person are:

**Complaints contact person**

| Name | Dr Kim Hansen |
| --- | --- |
| Position | Medical Director, Virtual Emergency Care Services, Metro North |
| Email | [Kim.Hansen@health.qld.gov.au](mailto:Kim.Hansen@health.qld.gov.au) |

| Name | Dr Andrew Staib |
| --- | --- |
| Position | Deputy Director Emergency Medicine, Princess Alexandra Hospital, Metro South |
| Email | Andrew.Staib@health.qld.gov.au |

| Name | Dr Andrew Hobbins King |
| --- | --- |
| Position | Medical Director Trauma and Senior Staff Specialist Emergency, Sunshine Coast University Hospital, Sunshine Coast Hospital and Health Service |
| Email | [Andrew.Hobbins-King@health.qld.gov.au](mailto:Andrew.Hobbins-King@health.qld.gov.au) |

If you have any complaints about any aspect of the project, the way it is being conducted or any questions about being a research participant in general, then you may contact the Chairperson on 07 4433 1440 or email [TSV-Ethics-Committee@health.qld.gov.au](mailto:TSV-Ethics-Committee@health.qld.gov.au).

**Consent Form**
*Adult providing own consent*

| **Title** | Digital intervention after whiplash injury |
| --- | --- |
| **Short Title** | Digital intervention after injury |
| **Protocol Number** | 1 |
| **Project Sponsor** | The University of Queensland |
| **Coordinating Principal Investigator/ Principal Investigator** | Dr Rachel Elphinston, PhD – RECOVER Injury Research Centre, The University of Queensland |
| **Associate Investigator(s)** | Prof Michele Sterling, PhD – RECOVER Injury Research Centre, The University of Queensland  Dr Nigel Armfield, PhD - RECOVER Injury Research Centre, The University of Queensland  A/Prof Clair Ashton-James, PhD – Pain Management Research Institute, University of Sydney  A/Prof Lisa Buckley, PhD – MAIC/UniSC Road Safety Research Collaboration, University of the Sunshine Coast  Dr Christopher Papic, PhD - RECOVER Injury Research Centre, The University of Queensland  Dr Anton Van Der Vegt, PhD – Centre for Health Services Research, The University of Queensland  Dr Katherine Brain, PhD – Chronic Brain Australia, University of Newcastle  Dr Kim Hansen, MBBS – Virtual Emergency Care Services, Metro North  Dr Andrew Staib, MBBS – Princess Alexandra Hospital, Metro South  Dr Andrew Hobbins King, BSc MBChB FACEM DCH – Sunshine Coast University Hospital, Sunshine Coast Hospital and Health Service  Bryce McMahon -- RECOVER Injury Research Centre, The University of Queensland  Jenna Liimatainen, BExPhys(Hons) -- RECOVER Injury Research Centre, The University of Queensland  Chloe-Emily Eather, BPsySci(Hons) – RECOVER Injury Research Centre, The University of Queensland |
| **Location** | The Virtual Emergency Care Service  Princess Alexandra Hospital  Sunshine Coast University Hospital |

**Declaration by Participant {Note that consent will be completed online by the participant}**

I have read the Participant Information Sheet or someone has read it to me in a language that I understand.

I understand the purposes, procedures and risks of the research described in the project.

I have had an opportunity to ask questions, and I am satisfied with the answers I have received.

I freely agree to participate in this research project as described and understand that I am free to withdraw at any time during the study without affecting my future health care.

By ticking this box, I acknowledge the above details and consent to participate in this study.

By ticking this box, I consent to be contacted to participate in future studies by the research team.

By ticking this box, I consent that the data I provide may be used to refine and further develop the intervention using large language models such as Enterprise Co-pilot. Please note that any data used will be completely non-identifiable (that is, there is no way anyone will be able to identify any details about you, or the information you provide) and only used for research and education purposes.

By ticking this box, I consent to for the research team to use this data for research training and education purposes. Please note that data used will be completely non-identifiable (that is, there is no way anyone will be able to identify any details about you, or the information you provide).

**Declaration by Researcher {Note that declaration will be completed online}**

I have answered any questions about the research project, and I believe that the participant has understood the purpose, procedures and risks involved.

| Researcher name (please print): |  |
| --- | --- |
| Date: |  |
| Researcher signature: |  |

**Form for Withdrawal of Participation**
*Adult providing own consent*

| **Title** | Digital intervention after whiplash injury |
| --- | --- |
| **Short Title** | Digital intervention after injury |
| **Protocol Number** | 1 |
| **Project Sponsor** | University of Queensland |
| **Coordinating Principal Investigator/ Principal Investigator** | Dr Rachel Elphinston, PhD – RECOVER Injury Research Centre, The University of Queensland |
| **Associate Investigator(s)** | Prof Michele Sterling, PhD – RECOVER Injury Research Centre, The University of Queensland  Dr Nigel Armfield, PhD - RECOVER Injury Research Centre, The University of Queensland  A/Prof Clair Ashton-James, PhD – Pain Management Research Institute, University of Sydney  A/Prof Lisa Buckley, PhD – MAIC/UniSC Road Safety Research Collaboration, University of the Sunshine Coast  Dr Christopher Papic, PhD - RECOVER Injury Research Centre, The University of Queensland  Dr Anton Van Der Vegt, PhD – Centre for Health Services Research, The University of Queensland  Dr Katherine Brain, PhD – Chronic Brain Australia, University of Newcastle  Dr Kim Hansen, MBBS – Virtual Emergency Care Services, Metro North  Dr Andrew Staib, MBBS – Princess Alexandra Hospital, Metro South  Dr Andrew Hobbins King, BSc MBChB FACEM DCH – Sunshine Coast University Hospital, Sunshine Coast Hospital and Health Service  Bryce McMahon -- RECOVER Injury Research Centre, The University of Queensland  Jenna Liimatainen, BExPhys(Hons) -- RECOVER Injury Research Centre, The University of Queensland  Chloe-Emily Eather, BPsySci(Hons) – RECOVER Injury Research Centre, The University of Queensland |
| **Location** | The Virtual Emergency Care Service  Princess Alexandra Hospital  Sunshine Coast University Hospital |

**Declaration by Participant**

I wish to withdraw from participation in the above research project and understand that such withdrawal will not affect my routine treatment, my relationship with those treating me or my relationship with the researchers, The University of Queensland, or Queensland Health.

|  | Name of Participant (please print) | |  |  |  |  |
| --- | --- | --- | --- | --- | --- | --- |
|  | | | | | | |
|  | Signature |  | | Date |  |  |
|  | | | | | | |

In the event that the participant’s decision to withdraw is communicated verbally, the Study Senior Researcher will need to provide a description of the circumstances below.
